# F17. DIFFERENCES IN INTRACRANIAL VOLUME, IQ AND PSYCHOPATHOLOGY IN YOUNG OFFSPRING OF PATIENTS AFFECTED WITH SCHIZOPHRENIA OR BIPOLAR DISORDER

**DOI:** 10.1093/schbul/sby017.548

**Published:** 2018-04-01

**Authors:** Neeltje van Haren, Setiaman Nikita, Martijn Koevoets, Heleen Baalbergen, Matthijs Vink, Esther Mesman, René Kahn, Manon Hillegers

**Affiliations:** 1 Brain Center Rudolf Magnus, University Medical Center Utrecht; 2 University Medical Center Utrecht; 3 Utrecht University; 4 Icahn School of Medicine at Mount Sinai; 5 Erasmus Medical Center, Sophia Children’s Hospital

## Abstract

**Background:**

Offspring of patients with schizophrenia and bipolar disorder are at increased risk to develop psychopathology. It has been suggested that the development of these disorders may be a result of early neurodevelopmental abnormalities. The intracranial volume (ICV) is a direct marker for neurodevelopment in the early years, as ICV reaches 90% of its full size around the age of five. Interestingly, a smaller ICV is more consistently found in SZ patients, compared to controls, than in bipolar disorder patients. The offspring of these two patients group may provide important information on the putative neurodevelopmental trajectory underlying the development of these disorders. We compared ICV between offspring of at least one parent with SZ (SZo) or BD (BDo) and control offspring (Co) in relation to IQ and the presence of psychopathology.

**Methods:**

A large sample of children and adolescents (8–18 years old; 54 SZo, 90 BDo, and 46 Co) was included. T1-weighted (3-Tesla) MRI brain scans were available for 146 participants. Group differences in ICV, global and local brain measures, psychopathology (K-SADS-PL, CBCL/6–18), IQ (WISC-III/WAIS-III), and their interactions were analyzed. FreeSurfer-5.3.0 was used for subcortical and cortical volume, cortical thickness, and cortical surface area estimations. Groups were compared using linear mixed effects modeling, corrected for family dependencies. FDR-correction was applied.

**Results:**

Our main finding was that ICV was significantly smaller in SZo, compared to BDo and Co. IQ was significantly lower in both SZo and BDo, relative to Co, but could not explain the smaller ICV in SZo. ICV was also not explained by psychopathology, even though there was no significant difference in ‘any psychopathology’ between SZo and BDo. There was however some illness specificity as BDo had a higher prevalence of ‘any mood disorder’ as compared with Co, and SZo had a higher prevalence of major depressive disorder and autism spectrum disorders as compared with BDo and Co. After correcting for ICV, the cortex was significantly thinner in SZo compared to BDo and Co, and BDo had larger lateral ventricles than Co. Without correction for ICV, volumes of the total brain and gray matter were significantly smaller in SZo than in BDo and Co. Cortical white matter volume was significantly smaller in SZo as compared with Co.

**Discussion:**

Irrespective of a lower IQ and increased presence of psychopathology in both high-risk offspring groups, abnormal early brain development, expressed as smaller ICV, differentiates offspring of patients with schizophrenia from offspring of patients with bipolar disorder and offspring of healthy parents. This suggest that the risk for schizophrenia is, in contrast to that in bipolar disorder, characterized by stunted brain development.

